# Stellate and Trabecular Keratitis

**Published:** 1888-02

**Authors:** 


### STELLATE AND TRABECULAR KERATITIS.

At the Congress of the French Association for the Advancement of Science, M. Gillet de Grandmont gave the following description of two new forms of keratitis. Keratitis in dotted furrows (*sillons etoilés*) is formed by diminutive sub-epithelial, star-shaped ulcerations. The rays of these ulcerations gradually develop into rectilinear furrows, forming serpiginous ulcerations, which occasionally produce sphacelus. Antiseptic treatment should be used. Trabecular keratitis is caused by the infiltration of the lymphatic cells into the corneal tubes, which present the appearance of small needles. This keratitis appears whenever the lymphatic circulation of the eyes is obstructed. The treatment consists in restoring the circulation by syndectomy, or by the evacuation of the aqueous humor. Trabecular keratitis may be either primary or secondary; it always follows kerectomy. In this case the trabeculæ are parallel with each other, and perpendicular with the curve of the fragment. They disappear as the wound cicatrizes.

Dr. Stœber (Nancy) made the following communication on the Binocular Convergent Function and the Metric Angle. Binocular vision is only possible where the different retinal images are perceived simultaneously. For this the two lines of vision must be parallel (in cases of emmetropia and absence of accommodation), and the distance between the center of the pupils at its maximum. When the object approaches the observer each eye must be optically adapted to the distance of the object. The two lines of vision must be directed on the object, in order to fuse the images produced in each eye into one perception. This direction of the lines of vision, which varies according to the distance between the eye and the object, is termed convergence; the relation between the two extreme points (infinity and the nearest point of distant binocular vision) is termed the power of convergence. Every line of vision directed to a near point makes a convergence or metric angle with the line of infinity. This angle is measured by Landolt's ophthalmo-dynamometer. Emmetropic, myopic or hypermetropic eyes placed in normal visual conditions, and directing their visual rays to a distance of one meter, have a metric angle which is equal to one; but the distance which separates the two rotary centres of the eyes, which is the basis line, must be measured. The convergent capacity is proportionate to the degree of accommodation, excepting in the case of extreme limits. It is greater in a myopic than in an emmetropic eye; it is weaker in a hypermetropic than in a normal eye.—*British Medical Journal.*

